# One-stop stroke management platform reduces workflow times in patients receiving mechanical thrombectomy

**DOI:** 10.3389/fneur.2022.1044347

**Published:** 2023-01-18

**Authors:** Tengfei Zhou, Tianxiao Li, Liangfu Zhu, Zhaoshuo Li, Qiang Li, Ziliang Wang, Liheng Wu, Yingkun He, Yucheng Li, Zhilong Zhou, Min Guan, Zhenkai Ma, Xiaoxi pei, Shuhui Meng, Yingpu Feng, Guifang Zhang, Wenli Zhao, Xiao Liu, Meiyun Wang

**Affiliations:** Henan Provincial People's Hospital, Zhengzhou, China

**Keywords:** stroke, time, puncture, thrombectomy, outcome

## Abstract

**Background and purpose:**

Clinical outcome in patients who received thrombectomy treatment is time-dependent. The purpose of this study was to evaluate the efficacy of the one-stop stroke management (OSSM) platform in reducing in-hospital workflow times in patients receiving thrombectomy compared with the traditional model.

**Methods:**

The data of patients who received thrombectomy treatment through the OSSM platform and traditional protocol transshipment pathway were retrospectively analyzed and compared. The treatment-related time interval and the clinical outcome of the two groups were also assessed and compared. The primary efficacy endpoint was the time from door to groin puncture (DPT).

**Results:**

There were 196 patients in the OSSM group and 210 patients in the control group, in which they were treated by the traditional approach. The mean DPT was significantly shorter in the OSSM group than in the control group (76 vs. 122 min; *P* < 0.001). The percentages of good clinical outcomes at the 90-day time point of the two groups were comparable (*P* = 0.110). A total of 121 patients in the OSSM group and 124 patients in the control group arrived at the hospital within 360 min from symptom onset. The mean DPT and time from symptom onset to recanalization (ORT) were significantly shorter in the OSSM group than in the control group. Finally, a higher rate of good functional outcomes was achieved in the OSSM group than in the control group (53.71 vs. 40.32%; *P* = 0.036).

**Conclusion:**

Compared to the traditional transfer model, the OSSM transfer model significantly reduced the in-hospital delay in patients with acute stroke receiving thrombectomy treatment. This novel model significantly improved the clinical outcomes of patients presenting within the first 6 h after symptom onset.

## Introduction

Thrombectomy recanalization of large-vessel occlusion in acute ischemic stroke is strongly time-dependent, and thus shortening the time between onset and recanalization can improve the prognosis of patients (1, 2). Hence, several pre-hospital and in-hospital measures for improvement have been implemented to shorten the time from onset to recanalization (ORT) (3, 4). In this regard, the development of a green channel that integrates medical specialists in multidisciplinary cooperation for hospital treatment of acute stroke considerably shortened the time from the door admission to recanalization treatment (5).

In most stroke centers, patients undergo imaging examination at an imaging center and are then transferred to the emergency department to receive intravenous thrombolysis and then transferred to the catheter room for thrombectomy. However, the long transfer times often cause delays in the treatment of patients with stroke. The one-stop stroke management (OSSM) platform combines computed tomography (CT), magnetic resonance imaging (MRI), and digital subtraction angiography (DSA) equipment in one space, using the same track to transfer patients from one device to another without switching beds. Thus, preoperative imaging examination and thrombectomy procedure are completed in the same space. Such an integrated combination of examination and treatment can dramatically shorten in-hospital transportation time. Our center, in collaboration with Siemens Healthcare, developed an OSSM platform to reduce the in-hospital delays of patients with acute stroke. In this study, we compared the data of patients who received thrombectomy *via* the OSSM platform with those who underwent the procedure *via* the traditional workflow to establish the effect of the OSSM platform model on the reduction of the delay of in-hospital stroke treatment.

## Methods

The data of patients who received thrombectomy through the OSSM platform and those of patients who underwent thrombectomy through the protocol transshipment pathway, defined as the control group, were retrospectively analyzed.

### Inclusion criteria

All the included patients received thrombectomy treatment at our center between January 2017 and December 2021. The data were retrieved from a prospectively maintained database. The inclusion criteria were as follows: (1) aged ≥18 years; (2) treated within 24 h from onset; (3) the National Institutes of Health Stroke Scale (NIHSS) ≥6; (4) Alberta Stroke Program Early Computed Tomography Score (ASPECT) ≥6; (5) pre-stroke Modified Rankin Scale (mRS) score ≤1; (6) the treatment protocols were consistent with those in the OSSM group or the control group; (7) the follow-up data were completed; and (8) informed consent was received from the patient or a family member.

### Treatment protocol

**Figure 1:**
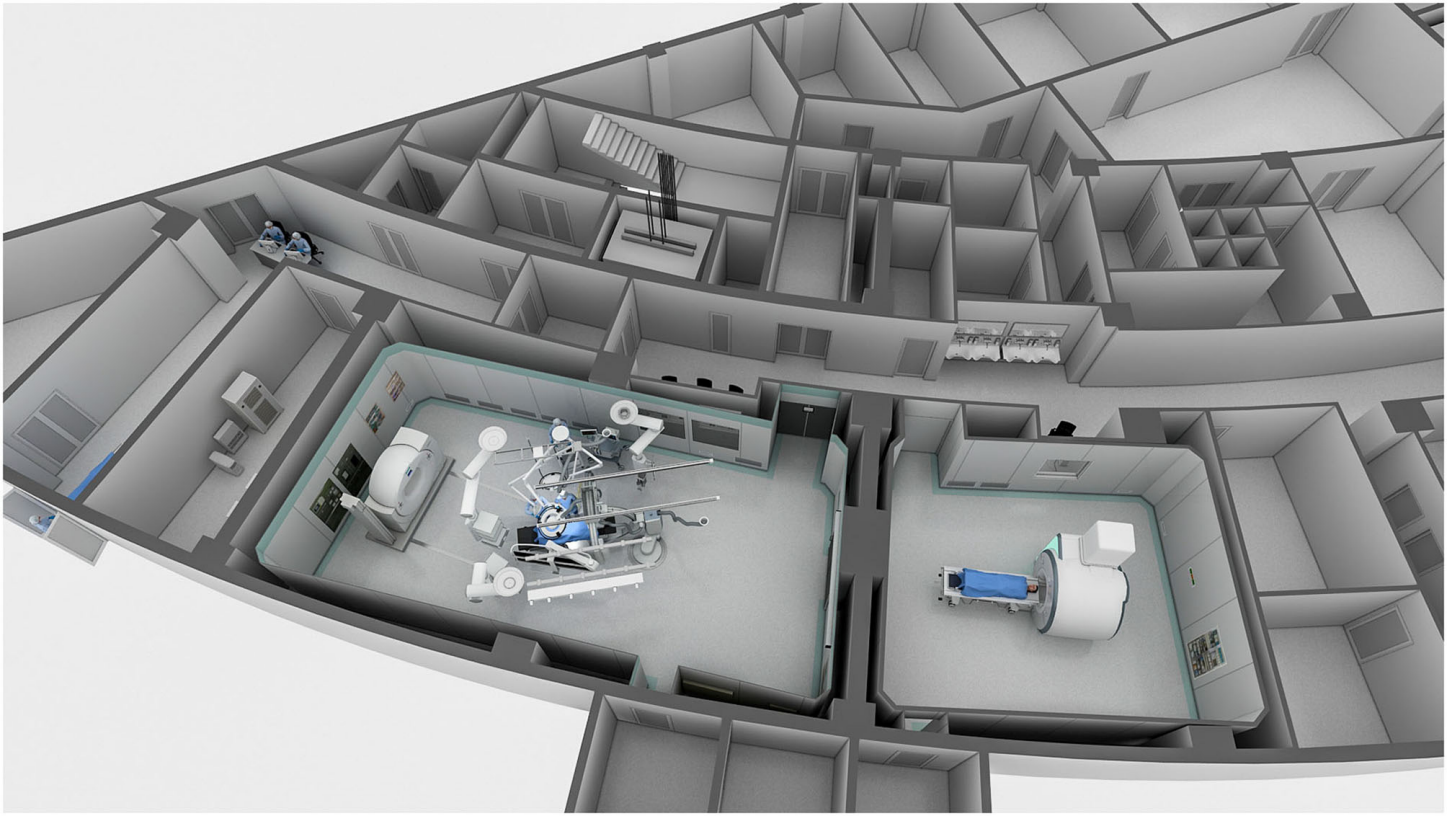
This overview of the one-stop stroke management (OSSM) platform shows the layout of the computed tomography (CT), magnetic resonance imaging (MRI), and digital subtraction angiography (DSA) equipment rooms.

In our center, patients with acute stroke typically arrive at the emergency department are then evaluated by a stroke physician, and receive the necessary treatment. The patients in the OSSM group were transferred to the OSSM platform for imaging examination, followed by thrombectomy. Patients who met the criteria of intravenous thrombolytic therapy (IVT) received IVT in the platform before thrombectomy therapy (Figure 1). Patients in the traditional protocol transshipment pathway group were transported from the emergency department to the imaging center for pretreatment imaging examination. Which imaging modality to choose was a comprehensive consideration based on the patient's actual condition, including the time from onset, severity, and the presence or absence of contraindications. Imaging examinations included noncontrast CT, CTP, MRI, and PWI, as well as noninvasive vascular imaging including CTA and MRA. Patients were then transferred to the catheter unit for thrombectomy therapy. Mechanical thrombectomy was performed under local or general anesthesia; heparin was used selectively. Endovascular thrombectomy methods included stent retrieval, aspiration thrombectomy, balloon dilatation, stent implantation, or other treatments based on the specific subject's clinical conditions.

### Relevant efficacy observation and follow-up examinations

The primary efficacy endpoint was the time from door to groin puncture (DPT). The secondary efficacy endpoints were the time from imaging to groin puncture (IPT), the time from door to successful reperfusion (DRT) or final angiographic results, the time from symptom onset to successful reperfusion (ORT), and the time from groin puncture to successful reperfusion (PRT). The rate of successful reperfusion of target vessels (modified thrombolysis in cerebral infarction, mTICI ≥ 2b) was defined as successful reperfusion; the rate of good functional independence was defined as an mRS score from 0 to 2 for a postoperative follow-up period of 90 ± 14 days. Safety outcomes included the rate of symptomatic intracranial hemorrhage (sICH) and all-cause mortality during the follow-up period. The complications of sICH were defined as any form of intracranial hemorrhage with an increase in NIHSS score of ≥ 4 points during the perioperative period.

### Statistical analysis

Statistical analysis was performed using the SPSS version 22.0 software. Continuous data that met normal distribution were represented with interquartile range or means with SD, comparison between groups used independent sample *t-*test, whereas categorical data were expressed as several cases and percentages. *P* < 0.05 was considered to be statistically significant.

## Results

A total of 406 patients were included in the analysis, of whom 196 were in the OSSM group and 210 were in the control group. The mean age was 63 years, the mean NIHSS score was 15, and the mean ODT was 407 min. The baseline data of the two groups of patients are presented in Table 1. No statistically significant differences were observed between the baseline data of the two groups, such as the baseline NIHSS score, gender, ASPECT, occlusion location, and the time from symptom onset to hospital admission. Furthermore, no significant differences were detected in the risk factors, such as a history of hypertension, diabetes, stroke history, and atrial fibrillation.

**Table 1:** Comparison of data between the two groups of patients underwent thrombectomy therapy.

	**All (406)**	**OSSM group (196)**	**Control group (210)**	***P***
Age (y)	63.3 ± 12.5 63.5 (21–91)	63.5 ± 12.6 63.5 (30–91)	63.0 ± 12.5 63 (21–89)	0.529
Male	271 (66.75%)	129 (65.92%)	142 (67.62%)	0.752
Hypertension	247 (60.84%)	120 (61.22%)	127 (60.48%)	0.919
Diabetes	104 (25.62%)	50 (25.51%)	54 (25.71%)	1.000
Stroke history	88 (21.67%)	46 (23.47%)	42 (20%)	0.402
Smoking history	87 (21.43%)	43 (21.94%)	44 (20.95%)	0.810
History of atrial fibrillation	168 (41.38%)	81 (41.33%)	87 (41.43%)	0.100
Baseline NIHSS	15.2 ± 5.0 15 (6–32)	15.3 ± 5.2 15 (6–32)	15.2 ± 4.8 15 (6–32)	0.858
Intravenous thrombolysis	57 (14.04%)	25 (12.75%)	32 (15.24%)	0.479
ASPECT score	7.606 ± 0.98 (6–10)	7.597 ± 0.9953 8 (6–10)	7.614 ± 0.9678 8 (6–10)	0.859
General anesthesia	393 (96.80%)	191 (97.45%)	202 (96.19)	0.472
**Occlusion location**				
ICA	105 (25.86%)	52 (26.53%)	53 (25.24%)	0.821
MCA	173 (42.61%)	84 (42.86%)	89 (42.38%)	1.000
ICA-MCA	40 (9.85%)	21 (10.71%)	19 (9.05%)	0.303
VA-BA	88 (21.67%)	39 (19.90%)	49 (23.33%)	0.470
**Time intervals (min)**				
ODT	406.6 ± 289.2	407.3 ± 297.2	405.9 ± 282.2	0.961
DPT	99.8 ± 3 0.5 99 (32–236)	76.4 ± 20.7 76 (32–191)	121.6 ± 20.2 121 (81–236)	0.001
DIT	32.9 ± 10.9 32 (13–88)	29.8 ± 11.0 28 (13–88)	35.8 ± 10.0 34 (19–76)	0.000
IPT	66.9 ± 28.3 63 (13–211)	46.7 ± 18.2 43 (13–121)	85.8 ± 22.3 85.5 (39–211)	0.000
PRT	88.7 ± 44.0 79 (15–320)	88.2 ± 43.7 78.5 (15–320)	89.3 ± 44.4 79 (15–320)	0.801
DRT	189.1 ± 52.8 188 (80–431)	164.6 ± 48.2 156.5 (80–396)	211.9 ± 46.4 202 (116–431)	0.000
ODT	406.6 ± 289.2 300 (30–1,440)	407.3 ± 297.2 300 (30–1,440)	405.9 ± 282.2 300 (30–1,440)	0.961
ORT	595.7 ± 292.1 496.5 (196–1,726)	571.9 ± 299.7 471.5 (196–1,726)	617.9 ± 283.8 526 (230–1,639)	0.114
mTICI2b/3	365 (89.90%)	178 (90.82%)	187 (89.05%)	0.554
mRS (0-2) at 90 days	176 (43.35%)	93 (47.45%)	83 (39.52%)	0.110
sICH	37 (9.11%)	16 (8.16%)	21 (10%)	0.606
Mortality	76 (18.72%)	34 (17.35%)	42 (20%)	0.526

ODT, time from symptom onset to door; DPT, time from door to groin puncture; DIT, time from door to imaging; IPT, time from imaging to groin puncture; PRT, time from groin puncture to reperfusion; DRT, time from door to reperfusion; ODT, time from symptom onset to door; ORT, time from symptom onset to reperfusion.

NIHSS, The National Institutes of Health Stroke Scale; ASPECT, Alberta Stroke Program Early Computed Tomography Score; ICA, Internal carotid artery; MCA, Middle cerebral artery, VA, vertebral artery; BA, Basilar artery. mTICI, modifified thrombolysis in cerebral infarction; sICH, symptomatic intracranial hemorrhage; mRS, modified Rankin scale.

The mean DPT was 76 min in the OSSM group and 122 min in the control group (*P* < 0.001). Notably, a 44 min reduction was observed in the OSSM group compared to the control group. The median DIT was significantly shorter in the OSSM group than in the control group (30 vs. 36 min; *P* < 0.001). The mean DPT was 47 in the OSSM group, whereas it was 86 min in the control group (*P* < 0.001). The mean DRT was 165 min in the OSSM group, whereas it was 212 min in the control group, and the difference between the two groups was statistically significant (*P* < 0.001). The mean PRT was comparable between the two groups (88 vs. 89 min; *P* = 0.701). The mean ORT was shorter in the OSSM group (572 min) than in the control group (618 min) but with no significant difference between the two groups (*P* = 0.114).

There was no significant between-group difference in the rate of successful reperfusion (90.82 vs. 89.05%, *P* = 0.554). The percentages of good clinical outcomes at the 90-day follow-up examination were 47% in the OSSM group and 40% in the control group. No statistical differences were present between the two groups (*P* = 0.110). The rate of all-cause 90-day mortality was 17% in the OSSM group and 20% in the control group (*P* = 0.526), the rate of symptomatic hemorrhage was 8% in the OSSM group and 10% in the control group, and the rate did not differ significantly across groups in pairwise comparisons (*P* = 0.606).

### Subgroup analyses

A total of 245 patients arrived at the hospital within 360 min from symptom onset, of whom 121 were in the OSSM group and 124 were in the control group (Table 2). There were no statistically significant differences in the baseline data between the two groups. Their mean ODT and PRT were comparable. In the OSSM group, the mean DPT, IRT, DRT, and ORT were significantly shorter than those in the control group. There was no significant between-group difference in the rate of successful reperfusion (90.82 vs. 89.05%, *P* = 0.554. A higher rate of good functional outcomes was achieved in the OSSM group than in the control group (53.71 vs. 40.32% *P* = 0.036). The rate of all-cause 90-day mortality and sICH were comparable between the two groups.

**Table 2:** Comparison of data between the two groups of patients underwent thrombectomy therapy within 6h from symptom onset.

	**All (245)**	**OSSM group (121)**	**Control group (124)**	***P***
Age (y)	62.9 ± 12.7 63 (30–91)	63.9 ± 12.6 65 (30–91)	61.9 ± 12.7 62 (30–89)	0.205
Male	163 (66.53%)	78 (64.46%)	85 (68.55%)	0.498
Hypertension	149 (60.82%)	74 (61.15%)	75 (60.48%)	1.000
Diabetes	58 (23.67%)	28 (23.14%)	30 (24.19%)	0.846
Stroke history	52 (21.22%)	25 (20.67%)	27 (21.77%)	0.831
Smoking history	46 (18.78%)	21 (17.35%)	25 (20.16%)	0.574
History of atrial fibrillation	105 (42.86%)	51 (42.15%)	54 (43.55%)	0.825
Baseline NIHSS	15.2 ± 4.7 15 (6–32)	15.4 ± 5.1 15 (6–32)	15.0 ± 4.3 15.5 (6–32)	0.51
Intravenous thrombolysis	38 (15.51%)	17 (14.04%)	21 (16.94%)	0.533
ASPECT score	7.6 ± 1.0 8 (6–10)	7.7 ± 1.0 8 (6–10)	7.6 ± 1.0 8 (6–10)	0.305
General anesthesia	235 (95.92%)	117 (96.69%)	118 (95.16%)	0.544
**Occlusion location**				
ICA	73 (29.80%)	36 (29.75%)	37 (29.84%)	0.988
MCA	98 (40%)	50 (43.12%)	48 (38.71%)	0.676
ICA-MCA	20 (8.16%)	9 (7.44%)	11 (8.87%)	0.682
VA-BA	54 (22.04%)	26 (21.49%)	28 (22.58%)	0.837
**Time intervals (min)**				
ODT	214.7 ± 82.7 240 (30–360)	216.2 ± 83.7 240 (30–360)	213.3 ± 82.0 240 (30–360)	0.782
DPT	99.6 ± 31.9 99 (36–236)	76.8 ± 23.6 76 (36–191)	121.8 ± 21.6 121 (90-236)	0.000
DIT	32.6 ± 11.2 32 (13–88)	29.3 ± 11.3 27 (13–88)	35.8 ± 10.2 31 (21–76)	0.000
IPT	67.0 ± 29.1 63 (13–211)	67.0 ± 29.1 44 (13–121)	86.0 ± 23.2 84 (39–211)	0.000
PRT	88.1 ± 41.8 79 (12–320)	87.2 ± 41.6 79 (15–320)	88.9 ± 42.1 81 (12-220)	0.762
DRT	188.5 ± 52.3 186 (81–369)	164.0 ± 46.9 156 (81–369)	212.3 ± 46.1 202 (116–320)	0.000
ODT	212.3 ± 46.1 240 (30–360)	216.2 ± 83.7 240 (30–360)	213.3 ± 82.0 240 (30–360)	0.782
ORT	403.2 ± 93.9 406 (169–619)	380.2 ± 91.9 381 (169–587)	425.6 ± 90.7 436 (230–619)	0.000
mRS (0–2) at 90 days	115 (46.94%)	65 (53.71%)	50 (40.32%)	0.036
sICH	23 (9.39%)	11 (9.09%)	12 (9.68%)	0.875
Mortality	40 (16.33%)	18 (14.88%)	22 (17.74%)	0.544

ODT, time from symptom onset to door; DPT, time from door to groin puncture; DIT, time from door to imaging; IPT, time from imaging to groin puncture; PRT, time from groin puncture to reperfusion; DRT, time from door to reperfusion; ODT, time from symptom onset to door; ORT, time from symptom onset to reperfusion.

NIHSS, The National Institutes of Health Stroke Scale; ASPECT, Alberta Stroke Program Early Computed Tomography Score; ICA, Internal carotid artery; MCA, Middle cerebral artery, VA, vertebral artery; BA, Basilar artery. mTICI, modifified thrombolysis in cerebral infarction; sICH, symptomatic intracranial hemorrhage; mRS, modified Rankin scale.

## Discussion

This retrospective cohort study showed that the OSSM platform incorporating CT, MRI, and DSA equipment can significantly reduce in-hospital delays in the thrombectomy treatment of patients with stroke compared to traditional workflow. The patients who arrived in the OSSM platform of the hospital within 6 h from symptom onset had a significantly shorter DPT and a better clinical outcome than those in the traditional protocol transshipment pathway.

Early ischemic brain reperfusion is critical for achieving good clinical outcomes in patients with stroke. A meta-analysis of five randomized clinical trials showed that of every 1,000 patients in whom substantial endovascular reperfusion was achieved; for every 15-min faster emergency department door-to-reperfusion time, an estimated 39 patients would have had a less-disabled outcome at 3 months (1). In the ESCAPE trial, the shorter imaging-to-reperfusion time significantly improved the chance of achieving a functionally independent outcome (6). A number of measures have been taken to reduce delays in the treatment of patients with stroke, particularly in improving the process of in-hospital imaging examination (7, 8).

Compared with multidetector CT, the latest generation of flat-panel CT (FPCT) is of high diagnostic value in the detection of ischemic changes. In addition, occluded vessels and cerebral collaterals can be detected with FP-CTA, and make it possible to be used as a peri-interventional diagnostic tool. Bypassing the CT suite, with direct transfer to angiosuite (DTAS), has been implemented in many centers and has contributed to a significant reduction in hospital workflow times (9). Marios-Nikos reported the case of a first mothership patient who was transported directly to the angiography suite and received nonenhanced FPCT (10). This patient was diagnosed with large-vessel occlusion, based on the FPCT-angiography results, and treated by endovascular thrombectomy, with a door-to-groin time (DNT) reduction to 23 min (10). A prospective, randomized trial showed that patients with acute stroke who were directly transferred to the angiosuite (DTAS) and received noncontrast-enhanced FPCT had a significantly reduced time from stroke imaging to groin puncture (by ~7 min) in comparison with the CT transit (CTT) pathway (11). Another study showed that DTAS protocols significantly increased the odds ratio of achieving a favorable outcome (12).

However, it should be pointed out that patients with DTAS directly transported to the angiography suite and received a noncontrast enhanced FPCT as preoperative imaging examination but not a CT-angiography or perfusion imaging, and as a result, patients evaluated by FPCT did not have large-vessel occlusion. Mendez reported the cases of patients, *n* = 7/97 (7.2%), whose initial angiogram did not show a treatable occlusion (12). The study of Psychogios included patients with NIHSS score > 10, but large-vessel occlusion was detected in the angiograms of only 18 of 30 patients (13). In addition, blindly transferring patients directly to the angiography room for plate CT examination also interferes with the normal operation of the C-arm for patients undergoing endovascular treatment. In our study, we creatively integrated CT, MR, and C-arm into the same space, and noninvasive vascular examinations such as CTA and MRA were performed to determine whether the patients were suitable for thrombectomy. For patients beyond 6 h from symptom onset, multimodal imaging was to be employed for treatment guidance, which was hard to be completed by FPCT.

In the present study, the OSSM model significantly shortened DPT compared to the traditional model. However, the time from admission to treatment was still long in comparison with those reported in previous publications, since more than 97% of the patients received general anesthesia during the operation, which requires a longer time than local anesthesia. For the majority of the patients, the preoperative imaging evaluation of patients in our center was based mainly on MR results, and thus a longer time was needed to complete an MR scan than a CT examination. This was also one of the main reasons that might have led to a longer delay.

It should be pointed out that with the extension of the thrombectomy time window to 24 h (14, 15), part of the thrombectomy patients arrived in the hospital beyond the 6-h time point. In this investigation, the median time from symptom onset to hospital admission was 6.7 h. The model described here did not significantly shorten the time from symptom onset to treatment; it did not significantly improve the clinical outcomes of the patients either. For patients who arrive at the hospital for treatment within a short time from onset, the OSSM transport mode is more likely to reduce the time from symptom onset to treatment, which can improve the prognosis of these patients compared with those in whom the traditional transport mode has been implemented.

Our study has some limitations. First of all, this was a retrospective analysis and comparison with a retrospective patient cohort. Certain selection biases in the baseline data might have been present, although the primary baseline data did not differ significantly between the two groups. Second, the study was carried out in a single center, and the equipment distribution, personnel allocation, and patient treatment process might have been different from those employed in other centers. Thus, the results of this study may not be applicable to other centers.

In conclusion, compared to the traditional transfer model, the OSSM platform significantly reduced the in-hospital delay in patients with acute stroke who received thrombectomy treatment. Furthermore, this model significantly improved the clinical outcomes in patients presenting within the first 6 h after symptom onset.

## Data availability statement

The raw data supporting the conclusions of this article will be made available by the authors, without undue reservation.

## Ethics statement

The studies involving human participants were reviewed and approved by Ethics Committee of Henan Provincial People's Hospital. Written informed consent for participation was not required for this study in accordance with the national legislation and the institutional requirements.

## Author contributions

TZ: conception and design, drafting the article, and revising it critically for important intellectual content. TL: conception and design and final approval of the version to be published. LZ, ZL, QL, ZW, LW, YH, YL, ZZ, MG, ZM, Xp, SM, YF, GZ, WZ, XL, and MW: acquisition of data, analysis, and interpretation of data. All authors agreed to be accountable for all aspects of the work in ensuring that questions related to the accuracy or integrity of any part of the work are appropriately investigated and resolved. All authors contributed to the article and approved the submitted version.
